# The environmental profile of a community’s health: a cross-sectional study on tobacco marketing in 16 countries

**DOI:** 10.2471/BLT.15.155846

**Published:** 2015-12-01

**Authors:** Emily Savell, Anna B Gilmore, Michelle Sims, Prem K Mony, Teo Koon, Khalid Yusoff, Scott A Lear, Pamela Seron, Noorhassim Ismail, K Burcu Tumerdem Calik, Annika Rosengren, Ahmad Bahonar, Rajesh Kumar, Krishnapillai Vijayakumar, Annamarie Kruger, Hany Swidan, Rajeev Gupta, Ehimario Igumbor, Asad Afridi, Omar Rahman, Jephat Chifamba, Katarzyna Zatonska, V Mohan, Deepa Mohan, Patricio Lopez-Jaramillo, Alvaro Avezum, Paul Poirier, Andres Orlandini, Wei Li, Martin McKee, Sumathy Rangarajan, Salim Yusuf, Clara K Chow

**Affiliations:** a Department for Health, University of Bath, Bath, England.; b Division of Epidemiology and Population Health, St John's Medical College and Research Institute, Bangalore, India.; c Population Health Research Institute, Hamilton Health Sciences and McMaster University, Hamilton, Canada.; d Faculty of Medicine, Universiti Teknologi MARA, Shah Alam, Malaysia.; e Faculty of Health Sciences, Simon Fraser University, Burnaby, Canada.; f Department of Internal Medicine, Universidad de La Frontera, Temuco, Chile.; g Department of Community Health, Universiti Kebangsaan Malaysia, Kuala Lumpur, Malaysia.; h Faculty of Health Sciences, Marmara University, Istanbul, Turkey.; i Sahlgrenska Academy, University of Gothenburg, Gothenburg, Sweden.; j Cardiovascular Research Institute, Isfahan University of Medical Sciences, Isfahan, Islamic Republic of Iran.; k School of Public Health, Post-Graduate Institute of Medical Education and Research, Chandigarh, India.; l Dr Somervell Memorial CSI Medical College, Karakonam, India.; m Africa Unit for Transdisciplinary Health Research, North-West University, Potchefstroom, South Africa.; n Primary Health Care Sector, Dubai Health Authority, Dubai, United Arab Emirates.; o Fortis Escorts Hospital, Jaipur, India.; p School of Public Health, University of the Western Cape, Cape Town, South Africa.; q Community Health Sciences, Aga Khan University, Karachi, Pakistan.; r Independent University Bangladesh, Dhaka, Bangladesh.; s Physiology Department, University of Zimbabwe College of Health Sciences, Harare, Zimbabwe.; t Department of Social Medicine, Wroclaw Medical University, Wroclaw, Poland.; u Madras Diabetes Research Foundation, Chennai, India.; v Medical School, Universidad de Santander, Bucaramanga, Colombia.; w Research Division, Dante Pazzanese Institute of Cardiology, São Paulo, Brazil.; x Institut Universitaire de Cardiologie et de Pneumologie de Québec, Québec, Canada.; y ECLA Foundation, Rosario, Santa Fe, Argentina.; z National Center for Cardiovascular Diseases, Chinese Academy of Medical Sciences, Beijing, China.; aa ECOHOST, London School of Hygiene & Tropical Medicine, London, England.; bb The George Institute for Global Health, Sydney Medical School (Westmead Campus), University of Sydney, PO Box M201, Missenden Road, Camperdown, NSW 2050, Australia.

## Abstract

**Objective:**

To examine and compare tobacco marketing in 16 countries while the Framework Convention on Tobacco Control requires parties to implement a comprehensive ban on such marketing.

**Methods:**

Between 2009 and 2012, a kilometre-long walk was completed by trained investigators in 462 communities across 16 countries to collect data on tobacco marketing. We interviewed community members about their exposure to traditional and non-traditional marketing in the previous six months. To examine differences in marketing between urban and rural communities and between high-, middle- and low-income countries, we used multilevel regression models controlling for potential confounders.

**Findings:**

Compared with high-income countries, the number of tobacco advertisements observed was 81 times higher in low-income countries (incidence rate ratio, IRR: 80.98; 95% confidence interval, CI: 4.15–1578.42) and the number of tobacco outlets was 2.5 times higher in both low- and lower-middle-income countries (IRR: 2.58; 95% CI: 1.17–5.67 and IRR: 2.52; CI: 1.23–5.17, respectively). Of the 11 842 interviewees, 1184 (10%) reported seeing at least five types of tobacco marketing. Self-reported exposure to at least one type of traditional marketing was 10 times higher in low-income countries than in high-income countries (odds ratio, OR: 9.77; 95% CI: 1.24–76.77). For almost all measures, marketing exposure was significantly lower in the rural communities than in the urban communities.

**Conclusion:**

Despite global legislation to limit tobacco marketing, it appears ubiquitous. The frequency and type of tobacco marketing varies on the national level by income group and by community type, appearing to be greatest in low-income countries and urban communities.

## Résumé

**Objectif:**

Examiner et comparer les pratiques de marketing du tabac dans 16 pays, alors que la Convention-cadre pour la lutte antitabac exige aux parties d'instaurer une interdiction globale de ce type de pratiques.

**Méthodes:**

De 2009 à 2012, des enquêteurs qualifiés ont rencontré 462 communautés, réparties dans 16 pays, le long d'un parcours d'un kilomètre afin de recueillir des données sur le marketing du tabac. Nous avons interrogé des membres de ces communautés au sujet de leur exposition aux formes traditionnelles et non traditionnelles de marketing dans les six mois précédents. Nous avons utilisé des modèles de régression multiniveaux permettant de contrôler les facteurs de confusion potentiels pour examiner les différences des pratiques de marketing entre les communautés urbaines et rurales ainsi qu'entre les pays à revenu élevé, intermédiaire et faible.

**Résultats:**

Le nombre de publicités pour le tabac observé dans les pays à revenu faible était 81 fois plus important que dans les pays à revenu élevé (rapport des taux d'incidence, RTI: 80,98; intervalle de confiance (IC) de 95%: 4,15–1578,42) et le nombre de points de vente de tabac était 2,5 fois plus élevé dans les pays à revenu faible et à revenu intermédiaire, tranche inférieure (RTI: 2,58; IC 95%: 1,17–5,67 et RTI: 2,52; IC: 1,23–5,17, respectivement). Sur les 11 842 personnes interrogées, 1184 (10%) ont indiqué rencontrer au moins cinq formes de marketing du tabac. Selon leurs déclarations, l'exposition à au moins une forme de marketing traditionnelle était 10 fois plus importante dans les pays à revenu faible que dans les pays à revenu élevé (rapport des cotes: 9,77; IC 95%: 1,24-76,77). Pour presque toutes les mesures, l'exposition aux pratiques de marketing était sensiblement plus faible dans les communautés rurales que dans les communautés urbaines.

**Conclusion:**

En dépit de la législation mondiale visant à limiter les pratiques de marketing du tabac, celles-ci sont très répandues. À l'échelle nationale, leur fréquence et leur type varient en fonction des tranches de revenus et du type de communauté, étant plus importantes dans les pays à revenu faible et les communautés urbaines.

## Resumen

**Objetivo:**

Examinar y comparar la publicidad del tabaco en 16 países mientras el Convenio Marco de la OMS para el Control del Tabaco obliga a las partes a implementar una prohibición generalizada en este tipo de publicidad.

**Métodos:**

Entre 2009 y 2012, investigadores entrenados completaron una ruta kilométrica en 462 comunidades de 16 países para recopilar datos sobre la publicidad del tabaco. Se entrevistó a miembros de cada comunidad sobre su exposición a la publicidad tradicional y no tradicional durante los seis meses previos. Se utilizaron modelos de regresión en múltiples niveles que controlaran los posibles factores de confusión para examinar las diferencias en la publicidad entre las comunidades urbanas y rurales y entre los países de ingresos altos, medios y bajos.

**Resultados:**

En comparación con los países de ingresos altos, la cantidad de anuncios sobre tabaco encontrados fue 81 veces superior en los países de ingresos bajos (razón de tasas de incidencia, IRR: 80,98; intervalo de confianza, IC, del 95%: 4,15–1578,42) y el número de estancos era 2,5 veces superior tanto en los países de ingresos bajos como en los países de ingresos medios más bajos (IRR: 2,58 (IC del 95%: 1,17–5,67 e IRR: 2,52; IC: 1,23-5,17, respectivamente). De los 11.842 entrevistados, 1.184 (10%) informaron haber visto al menos cinco tipos de publicidad del tabaco. La exposición autodeclarada a al menos una clase de publicidad tradicional fue 10 veces más alta en los países de ingresos bajos que en los países de ingresos altos (cociente de posibilidades: 9,77 (IC del 95%: 1,24–76,77). En prácticamente todas las mediciones, la exposición era significativamente más baja en las comunidades rurales que en las comunidades urbanas.

**Conclusión:**

A pesar de la legislación global para limitar la publicidad del tabaco, esta parece ubicua. La frecuencia y la clase de publicidad del tabaco varían en un nivel nacional por grupo de ingresos y tipo de comunidad, y parece ser mayor en los países de ingresos bajos y en las comunidades rurales.

## ملخص

**الغرض:**

دراسة تسويق التبغ ومقارنته في 16 دولة في الوقت الذي تتطلب فيه الاتفاقية الإطارية بشأن مكافحة التبغ وجود أطراف تعمل على تطبيق حظر شامل لتسويق هذا النوع من المنتجات.

**الطريقة:**

أكمل الباحثون المدربون في الفترة بين عامي 2009 و2012 مسيرة تبلغ كيلومترًا على الأقدام في 462 من المجتمعات المحلية في 16 دولة لجمع البيانات عن تسويق التبغ. وأجرينا مقابلات مع أعضاء هذه المجتمعات تناولت مدى تعرضهم للطرق التقليدية وغير التقليدية لهذا التسويق خلال الستة أشهر الماضية. وقد استخدمنا نماذج التحوّف متعددة المستويات مع التحكم في العوامل المحيّرة المحتملة لدراسة الاختلافات في التسويق بين المجتمعات الريفية والحضرية، والفروق بين الدول مرتفعة الدخل ومتوسطة الدخل ومنخفضة الدخل.

**النتائج:**

زاد عدد الإعلانات التجارية للتبغ التي تضمنتها الملاحظة في الدول منخفضة الدخل مقارنةً بالدول مرتفعة الدخل، حيث بلغت الزيادة 81 ضعفًا (نسبة معدل وقوع الحالة (IRR)‏: 80.98؛ بنسبة أرجحية مقدارها 95%: 4.15 – 1578.42) وكان عدد منافذ بيع التبغ أعلى في كل من الدول منخفضة الدخل والدول متوسطة الدخل من الشريحة الدنيا (‏IRR: 2.58؛ بنسبة أرجحية مقدارها 95%: 1.17 – 5.67 وIRR: 2.52؛ بنسبة أرجحية: 1.23 – 5.17، على التوالي). وذكر 1184 (10%) ممن تم إجراء المقابلة معهم والذين بلغ عددهم 11,842 أنهم شاهدوا خمسة أنواع على الأقل من وسائل تسويق التبغ. وزاد عدد حالات الإبلاغ الذاتي عن التعرض لنوع واحد على الأقل من أساليب التسويق التقليدية، بحيث بلغت الزيادة 10 أضعاف العدد في الدول مرتفعة الدخل وذلك في الدول منخفضة الدخل (بنسبة احتمال: 9.77؛ ونسبة أرجحية مقدارها 95%: 1.24–76.77). وطبقًا لجميع القياسات تقريبًا، انخفضت نسبة التعرض لأساليب التسويق بدرجة ملحوظة في المجتمعات الريفية مقارنةً بالمجتمعات الحضرية.

**الاستنتاج:**

بالرغم من ****وجود ****تشريعات عالمية للحد ****من تسويق التبغ، يبدو أن هذا التسويق واسع الانتشار. وتختلف أنواع تسويق التبغ ووتيرته على المستوى المحلي للدول حسب فئة الدخل ونوع المجتمع المحلي، كما يبدو أنه يصل إلى أقصى مستوى له في الدول منخفضة الدخل والمجتمعات

## 摘要

**目的:**

旨在调查并比较 16 个国家烟草营销情况，而烟草控制框架公约要求各缔约方全面禁止此类营销。

**方法:**

在** **2009 年至 2012 年间，受过培训的调查员大量走访了 16 个国家的 462 个社区，收集有关烟草营销的数据。我们就社区居民过去六个月接受传统和非传统营销的情况对其进行了访问。为了调查城市和农村社区之间，以及高、中、低收入国家之间市场营销的差异，我们使用多层次回归模型控制潜在的混杂变量。

**结果:**

相对于高收入国家，低收入国家的烟草广告数量是其 81 倍（发病率之比，内部收益率 IRR：80.98，95% 置信区间 CI：4.15-1578.42），低收入和中低收入国家的烟草销售点数量高出 2.5 倍（内部收益率 IRR：2.58，95% 置信区间 CI：1.17–5.67，内部收益率 IRR：2.52，置信区间 CI：分别为 1.23–5.17）。在 11842 位受访者中，有 1184 位 (10％) 称看到过至少五类烟草营销。低收入国家自称受到至少一类传统营销的人数是高收入国家的 10 倍（比值：9.77，95% 置信区间 CI：1.24–76.77)。几乎所有营销手段对农村社区的影响显著低于城市社区。

**结论:**

尽管全球立法限制烟草营销，但它无处不在。由于收入情况和社区类型的差异，烟草营销的频率和类型在各国均有所差异，其中低收入国家和城市社区烟草营销最多。

## Резюме

**Цель:**

Изучить и сравнить маркетинг табака в 16 странах, принимая во внимание требование Рамочной конвенции Всемирной организации здравоохранения по борьбе против табака ввести полный запрет на маркетинг подобного рода в государствах-участниках.

**Методы:**

В период между 2009 и 2012 годами обученные исследователи проходили путь длиной в 1 км в 462 общинах 16 стран и собирали данные о маркетинге табака. Жители исследуемой общины опрашивались относительно того, приходилось ли им сталкиваться с традиционным и нетрадиционным маркетингом такого рода за последние шесть месяцев. Для изучения маркетинговых различий между городскими и сельскими общинами, а также для выявления различий между странами с низким, средним и высоким уровнем дохода были использованы модели многоуровневой регрессии с контролем потенциальных, искажающих результаты факторов.

**Результаты:**

По сравнению со странами, характеризующимися высоким уровнем дохода, в странах с низким уровнем дохода реклама табака наблюдалась в 81 раз чаще (отношение частоты случаев, ОЧС: 80,98; 95% доверительный интервал, ДИ: 4,15–1578,42), а количество торговых точек, реализующих табачные изделия, было в 2,5 раза больше в странах с низким уровнем дохода и уровнем дохода ниже среднего (ОЧС: 2,58; 95% ДИ: 1,17–5,67 и ОЧС: 2,52; ДИ: 1,23–5,17 соответственно). Из 11 842 опрошенных 1184 человека (10%) сообщили о том, что сталкивались по меньшей мере с пятью видами маркетинга табака. О контакте по меньшей мере с одним из традиционных видов маркетинга табака респонденты самостоятельно сообщали в 10 раз чаще в странах с низким доходом по сравнению со странами с высоким уровнем дохода (отношение шансов: 9,77; 95% ДИ: 1,24–76,77). Почти по всем показателям уровень маркетингового охвата в сельских общинах был значительно ниже, чем в городских.

**Вывод:**

Несмотря на то что мировое законодательство ограничивает маркетинг табака, он встречается повсеместно. Частота и тип маркетинга табака на национальном уровне зависят от уровня дохода и типа общины, причем эти показатели являются наиболее высокими для городских общин и стран с низким уровнем дохода.

## Introduction

Tobacco is a leading cause of morbidity and mortality, responsible for an estimated 18%, 11% and 4% of deaths in high-, middle- and low-income countries, respectively.^1^ Since the prevalence of smoking is falling in high-income countries but increasing in many middle- and low-income countries, the global burden of disease caused by tobacco use is expected to shift increasingly from high-income countries to countries with lower incomes.

As marketing by the tobacco industry plays a substantial role in smoking initiation,^2^^–^^4^ complete bans on such marketing can be an effective means of reducing tobacco use.^5^^,^^6^ In 2005, the World Health Organization’s (WHO’s) Framework Convention on Tobacco Control (FCTC) called for a comprehensive ban on all tobacco marketing.^7^ However, the lack of relevant capacity and/or political will in many countries and the insidious influence of the tobacco industry have meant that the implementation of some of the FCTC’s recommendations has been slow.^8^

In this paper, we assess the global tobacco marketing environment by examining and comparing the extent and nature of tobacco marketing in 462 communities spread across 16 low-, middle- and high-income countries.

## Methods

### Data source

All of the data we analysed were collected as part of the Environmental Profile of a Community’s Health study, which has already been described in detail.^9^^–^^11^ This study is a component of the Prospective Urban Rural Epidemiology study – a large cohort study that is designed to examine the relationship between lifestyle factors and cardiovascular disease in adults aged 35–70 years.^10^^,^^11^ The Environmental Profile of a Community’s Health study includes an objective environmental audit in which trained investigators walk a predefined kilometre-long route within a study community. During each such walk, the investigators visit stores and systematically record physical aspects of the environment – e.g. the number of tobacco advertisements that they see. The second part of the Environmental Profile of a Community’s Health study involves an interviewer-administered questionnaire that captures individuals’ perceptions of their community – including whether the interviewees recall seeing certain types of tobacco marketing within the previous six months.^9^ This questionnaire was administered to a subsample of the participants of the Prospective Urban Rural Epidemiology study.

We investigated data collected, between 2009 and 2012, in 16 countries. According to the World Bank’s 2006 classification,^11^ three of the countries – Canada, Sweden and the United Arab Emirates – were high-income, seven – Argentina, Brazil, Chile, Malaysia, Poland, South Africa and Turkey – were upper-middle-income, three – China, Colombia and the Islamic Republic of Iran – were lower-middle-income – and three – India, Pakistan and Zimbabwe – were low-income. Although Bangladesh is included in the Prospective Urban Rural Epidemiology study,^10^ we excluded Bangladeshi data on tobacco marketing from our analyses because they were relatively incomplete.

### Measures of marketing

The Environmental Profile of a Community’s Health study records both push and pull marketing. Push marketing, which aims to increase product availability,^12^^,^^13^ was measured by trained researchers who recorded the number of tobacco outlets – e.g. vendors, street stands and general stores – seen during the audit walk and whether a tobacco-selling store visited during the walk sold single cigarettes. Pull marketing, which encourages customers to seek out a product through advertising and promotion,^12^^,^^13^ was measured using both direct observation – i.e. the number of tobacco advertisements counted during the audit walk and whether the tobacco-selling store visited during the walk had point-of-sale tobacco advertising – and via self-report in interviews – i.e. whether an interviewee recalled seeing various forms of tobacco advertising in the previous six months. Almost all of the tobacco marketing measures that we examined reflected those covered by the FCTC^7^ or the associated implementation guidelines.^14^ However, we also assessed tobacco outlet density as this has been shown to play an important role in smoking prevalence among adults and adolescents.^15^^,^^16^

### Observed data

For each country and country income group, the mean numbers of tobacco outlets and advertisements observed per community, the percentage of visited stores that sold single cigarettes and the percentage of visited stores that had point-of-sale tobacco advertising, were calculated – separately for the urban and rural communities.

As statistical tests showed that our outcome data were highly overdispersed, we used negative binomial multilevel regression models to examine differences in the number of observed tobacco outlets and tobacco advertisements between urban and rural communities and between country income groups. In these models, the number of outlets or advertisements was used as the outcome variable. Country income group and community type – i.e. rural or urban – were used as the categorical explanatory variables, and a random effect was included for the country. Incidence rate ratios (IRRs) were obtained by exponentiation of the regression coefficient and reported with the corresponding 95% confidence intervals (CIs). As data on the sale of single cigarettes and point-of-sale advertising were based on only one tobacco-selling store per community – and it is not possible to know whether the selected store was representative of all tobacco-selling stores within the community – such data were not included in the regression analyses.

### Self-reported data

To examine differences in self-reported marketing levels between community types and across country income groups, we considered 13 binary outcome variables. These included whether or not individuals reported seeing tobacco marketing of any of six traditional types of media – i.e. posters, signage, television, radio, print and cinema – and five non-traditional types – i.e. sponsorship, marketing on other products, marketing on the internet, free samples and vouchers. We also combined all the traditional types and all the non-traditional types of marketing into two separate binary variables.

We applied a logistic multilevel regression model to each of the binary outcome measures and again included categorical explanatory variables for country income group and community type. We also included random effects for country and community. Each model was adjusted for potential confounders – i.e. sex, age, education, smoking status, having close friends who smoke, access to the internet, television ownership and radio ownership.^2^^,^^17^^–^^19^ The resulting odds ratios (ORs) are reported with corresponding 95% CIs.

All of the models were fitted using the glmmadmb and glmer functions from the glmmADMB and lme4 packages of R version 3.0.2 (R Foundation, Vienna, Austria).

## Results

We analysed data from 235 urban and 227 rural communities, across 16 countries (Table 1). Overall, 11 842 individuals who resided in the observed communities – i.e. 5809 in the urban and 6033 in the rural communities – were interviewed and included in the final analyses.

**Table 1:** Sample sizes for a tobacco marketing study in 462 communities, 16 countries, 2009–2012

Country^a^	No. of study communities				No. of interviewees		
	Total	Urban	Rural		Total	Urban	Rural
All	462	235	227		11 842	5809	6033
**High-income**							
Canada	46	31	15		1145	807	338
Sweden	23	20	3		580	496	84
United Arab Emirates	3	1	2		89	26	63
Total	72	52	20		1814	1329	485
**Upper-middle-income**							
Argentina	20	6	14		544	171	373
Brazil	14	7	7		387	202	185
Chile	5	2	3		127	51	76
Malaysia	33	18	15		1168	591	577
Poland	4	1	3		89	26	63
South Africa	6	3	3		194	99	95
Turkey	38	25	13		1207	795	412
Total	120	62	58		3716	1935	1781
**Lower-middle-income**							
China	101	39	62		3131	1224	1907
Colombia	54	31	23		278	151	127
Iran (Islamic Republic of)	20	11	9		593	321	272
Total	175	81	94		4002	1696	2306
**Low-income**							
India	88	37	51		2118	766	1352
Pakistan	4	2	2		111	57	54
Zimbabwe	3	1	2		81	26	55
Total	95	40	55		2310	849	1461

^a^ Countries were categorized according to the World Bank’s 2006 classification.^11^

### Observed data

#### Push marketing

**Fig. 1:**
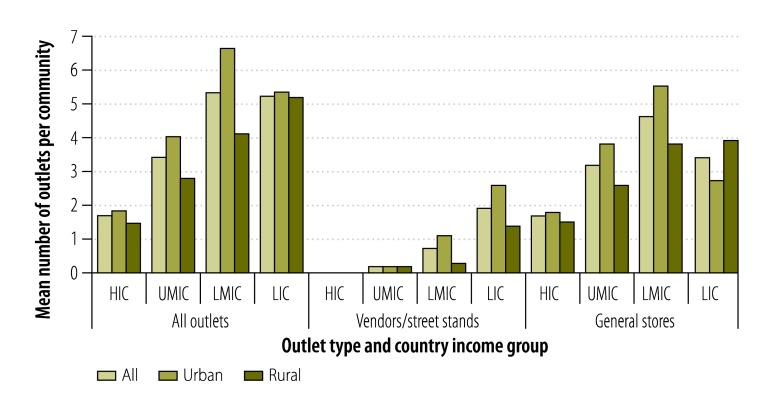
Tobacco-selling outlets in urban or rural study community, 16 countries, 2009–2012

HIC: high-income country; LIC: low-income country; LMIC: lower-middle-income country; UMIC: upper-middle-income country.

Notes: Countries were categorized according to the World Bank’s 2006 classification.^11^ Outlets were counted during a kilometre-long audit walk in each study community.

There were marked differences in outlet type and density between countries and country income group (Fig. 1 and Table 2 available at: http://www.who.int/bulletin/volumes/93/12/15-155846). The mean number of tobacco-selling outlets observed in each community increased with decreasing country income, from 1.7 in the high-income countries to 3.4 in the upper-middle-income countries and over 5.0 in the lower-middle-income and low-income countries. This trend was driven largely by the relatively high numbers of vendors and street stands observed – a mean of almost two per community – in low-income countries. No such outlets were observed in high-income countries and, on average, only 0.2 and 0.7 were observed per community in the upper-middle-income and lower-middle-income countries, respectively. The mean number of general stores observed per community did not follow the same pattern – 1.7, 3.2, 4.6 and 3.4 in the high-, upper-middle-, lower-middle- and low-income countries, respectively.

**Table 2:** Observed push and pull tobacco marketing, 16 countries, 2009–2012

Country^a^	Push marketing					Pull marketing	
	Outlets selling cigarettes/tobacco			No. of selected tobacco stores selling single cigarettes, (%)		Mean no. of cigarette or tobacco adverts	No. of selected tobacco stores with POS advertising, (%)
	Mean no. of outlets^b^	Mean no. of vendors or street stands	Mean no. of general stores				
**All countries**							
All communities (*n* = 462)	4.2	0.7	3.5	145/461 (31.5)		1.3	139/458 (30.4)
Urban (*n* = 235)	4.6	0.9	3.7	74/235 (31.5)		1.7	96/235 (40.9)
Rural (*n* = 227)	3.8	0.5	3.3	71/226 (31.4)		0.9	43/223 (19.3)
**High-income countries**							
Canada							
Urban (*n* = 31)	1.5	0.0	1.5	0/31 (0.0)		0.0	3/31 (9.7)
Rural (*n* = 15)	1.1	0.0	1.1	0/15 (0.0)		0.0	0/15 (0.0)
Sweden							
Urban (*n* = 20)	2.1	0.0	2.1	0/20 (0.0)		0.8	10/20 (50.0)
Rural (*n* = 3)	1.0	0.0	1.0	0/3 (0.0)		0.0	0/3 (0.0)
United Arab Emirates							
Urban (*n* = 1)	6.0	0.0	6.0	1/1 (100.0)		0.0	0/1 (0.0)
Rural (*n* = 2)	4.5	0.0	4.5	1/2 (50.0)		0.0	0/2 (0.0)
Total							
All communities (*n* = 72)	1.7	0.0	1.7	2/72 (2.8)		0.2	13/72 (18.1)
Urban (*n* = 52)	1.8	0.0	1.8	1/52 (1.9)		0.3	13/52 (25.0)
Rural (*n* = 20)	1.5	0.0	1.5	1/20 (5.0)		0.0	0/20 (0.0)
**Upper-middle-income countries**							
Argentina							
Urban (*n* = 6)	2.0	0.0	2.0	2/6 (33.3)		0.5	1/6 (16.7)
Rural (*n* = 14)	0.8	0.0	0.8	3/14 (21.4)		0.5	1/14 (7.1)
Brazil							
Urban (*n* = 7)	1.0	0.3	0.7	0/7 (0.0)		10.4	7/7 (100.0)
Rural (*n* = 7)	2.0	0.0	2.0	0/7 (0.0)		6.0	7/7 (100.0)
Chile							
Urban (*n* = 2)	3.0	1.0	2.0	0/2 (0.0)		0.5	1/2 (50.0)
Rural (*n* = 3)	1.3	0.3	1.0	2/3 (66.7)		1.0	3/3 (100.0)
Malaysia							
Urban (*n* = 18)	5.8	0.2	5.6	0/18 (0.0)		0.1	9/18 (50.0)
Rural (*n* = 15)	7.2	0.7	6.5	4/15 (26.7)		0.1	7/15 (46.7)
Poland							
Urban (*n* = 1)	8.0	0.0	8.0	0/1 (0.0)		0.0	0/1 (0.0)
Rural (*n* = 3)	1.3	0.0	1.3	0/3 (0.0)		0.0	0/3 (0.0)
South Africa							
Urban (*n* = 3)	3.3	1.0	2.3	1/3 (33.3)		0.0	1/3 (33.3)
Rural (*n* = 3)	1.3	0.0	1.3	1/3 (33.3)		0.0	1/3 (33.3)
Turkey							
Urban (*n* = 25)	4.0	0.0	4.0	0/25 (0.0)		0.1	9/25 (36.0)
Rural (*n* = 13)	1.2	0.0	1.2	0/13 (0.0)		0.1	1/13 (7.7)
Total							
All communities (*n* = 120)	3.4	0.2	3.2	13/120 (10.8)		1.1	48/120 (40.0)
Urban (*n* = 62)	4.0	0.2	3.8	3/62 (4.8)		1.3	28/62 (45.2)
Rural (*n* = 58)	2.8	0.2	2.6	10/58 (17.2)		0.9	20/58 (34.5)
**Lower-middle-income countries**							
China							
Urban (*n* = 39)	6.7	0.4	6.3	0/39 (0.0)		0.5	8/39 (20.5)
Rural (*n* = 62)	3.0	0.0	2.9	0/61 (0.0)		0.0	0/58 (0.0)
Colombia							
Urban (*n* = 31)	7.7	2.4	5.3	31/31 (100.0)		3.3	17/31 (54.8)
Rural (*n* = 23)	7.3	1.2	6.2	23/23 (100.0)		2.1	10/23 (43.5)
Iran (Islamic Republic of)							
Urban (*n* = 11)	3.0	0.0	3.0	7/11 (63.6)		0.0	0/11 (0.0)
Rural (*n* = 9)	3.9	0.0	3.9	8/9 (88.9)		0.1	1/9 (11.1)
Total							
All communities (*n* = 175)	5.3	0.7	4.6	69/174 (39.7)		1.0	36/171 (21.1)
Urban (*n* = 81)	6.6	1.1	5.5	38/81 (46.9)		1.5	25/81 (30.9)
Rural (*n* = 94)	4.1	0.3	3.8	31/93 (33.3)		0.6	11/90 (12.2)
**Low-income countries**							
India							
Urban (*n* = 37)	5.4	2.8	2.6	32/37 (86.5)		4.5	28/37 (75.7)
Rural (*n* = 51)	5.2	1.3	3.9	29/51 (56.9)		1.3	9/51 (17.7)
Pakistan							
Urban (*n* = 2)	4.0	0.0	4.0	0/2 (0.0)		3.0	1/2 (50.0)
Rural (*n* = 2)	3.0	0.0	3.0	0/2 (0.0)		8.0	1/2 (50.0)
Zimbabwe							
Urban (*n* = 1)	1.0	0.0	1.0	0/1 (0.0)		0.0	1/1 (100.0)
Rural (*n* = 2)	7.5	4.0	3.5	0/2 (0.0)		8.5	2/2 (100.0)
Total							
All communities (*n* = 95)	5.2	1.9	3.4	61/95 (64.2)		2.8	42/95 (44.2)
Urban (*n* = 40)	5.3	2.6	2.7	32/40 (80.0)		4.3	30/40 (75.0)
Rural (*n* = 55)	5.2	1.4	3.9	29/55 (52.7)		1.8	12/55 (21.8)

POS: point-of-sale.

^a^ Countries were categorized according to the World Bank’s 2006 classification.^11^

^b^ Includes vendors, street stands and general stores.

Note: Observed numbers by trained investigators during a kilometre-long walk in each community.

Combining data from all 16 countries, more vendors and/or street stands were observed in the urban communities than in the rural – means of 0.9 and 0.5 per community, respectively – and the urban communities also had a higher mean number of general stores selling tobacco – 3.7, compared with 3.3 per rural community. However, these urban/rural differences were not consistent across all four country income groups (Fig. 1 and Table 2).

After controlling for community type and country income group, the upper-middle-income countries had similar numbers of tobacco outlets (IRR: 1.29; 95% CI: 0.67–2.49) compared with high-income countries, but lower-middle-income countries (IRR: 2.52; 95% CI: 1.23–5.17) and low-income countries (IRR: 2.58; 95% CI: 1.17–5.67) had significantly more (Table 3). Across all countries, the mean number of tobacco outlets observed per community was significantly lower in rural than in urban communities (IRR: 0.73; 95% CI: 0.63–0.85; Table 3).

**Table 3:** Incidence rate ratios for push and pull observed marketing of tobacco, 16 countries, 2009–2012

Group	IRR (95% CI)^a^	
	Tobacco outlets^b^	Tobacco advertisements^b^
**Community type**		
Urban	1	1
Rural	0.73 (0.63–0.85)	0.40 (0.26–0.60)
**Country income group^c^**		
High	1	1
Upper-middle	1.29 (0.67–2.49)	3.96 (0.30–52.88)
Lower-middle	2.52 (1.23–5.17)	4.68 (0.26–85.00)
Low	2.58 (1.17–5.67)	80.98 (4.15–1578.42)

CI: confidence interval; IRR: incidence rate ratio.

^a^ Derived from negative binomial multilevel regression models.

^b^ Based on the mean numbers of outlets and advertisements observed during a kilometre-long audit walk in each community.

^c^ Countries were categorized according to the World Bank’s 2006 classification.^11^

**Fig. 2:**
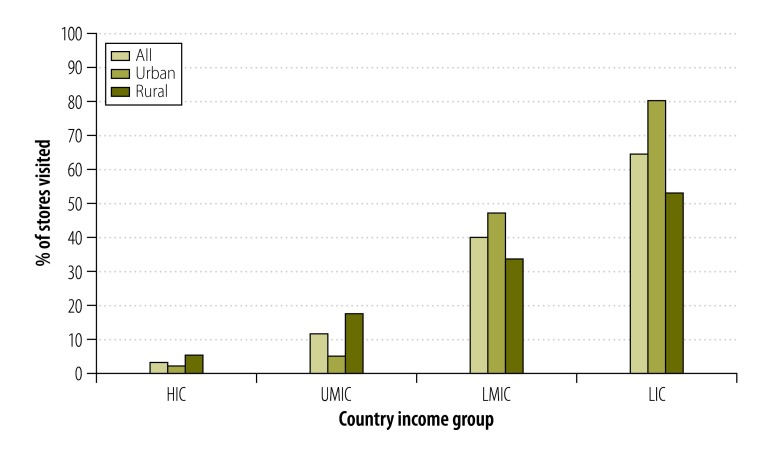
Proportion of tobacco-selling stores selling single cigarettes, 16 countries, 2009–2012

HIC: high-income country; LIC: low-income country; LMIC: lower-middle-income country; UMIC: upper-middle-income country.

Note: Countries were categorized according to the World Bank’s 2006 classification.^11^

The sale of single cigarettes was not observed in any of the communities in eight of the countries (Table 2). However, overall, outlets selling single cigarettes became increasingly common with declining country income (Fig. 2 and Table 2). Although the urban/rural differences in the sale of single cigarettes varied by country income group, the sale of single cigarettes was more common in urban than rural communities in both lower-middle- and low-income countries.

#### Pull marketing

**Fig. 3:**
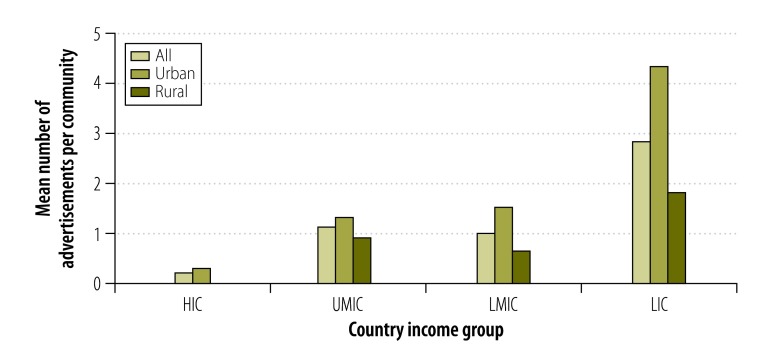
Tobacco advertisements in urban or rural study community, 16 countries, 2009–2012

HIC: high-income country; LIC: low-income country; LMIC: lower-middle-income country; UMIC: upper-middle-income country.

Notes: Countries were categorized according to the World Bank’s 2006 classification.^11^ Advertisements were counted during a kilometre-long audit walk in each study community.

Tobacco advertisements were much more common in low-income countries than in the other countries. Very few tobacco advertisements were seen in high-income countries. In middle- and low-income countries, means of approximately 1 and 3 observed advertisements per community were recorded, respectively (Fig. 3 and Table 2). Combining data from all countries, tobacco advertisements were more common in the urban than rural communities, with means of 1.7 and 0.9 observed per community, respectively.

After controlling for community type and country income group, the middle-income countries had similar numbers of tobacco advertisements (upper-middle-income IRR: 3.96; 95% CI: 0.30–52.88 and lower-middle-income IRR: 4.68; 95% CI: 0.26–85.00) as the high-income countries, whereas low-income countries had many more (IRR: 80.98; 95% CI: 4.15–1578.42). Overall, the mean number of tobacco advertisements observed per community was much lower in rural communities than in urban communities (IRR: 0.40; 95% CI: 0.26–0.60; Table 3).

**Fig. 4:**
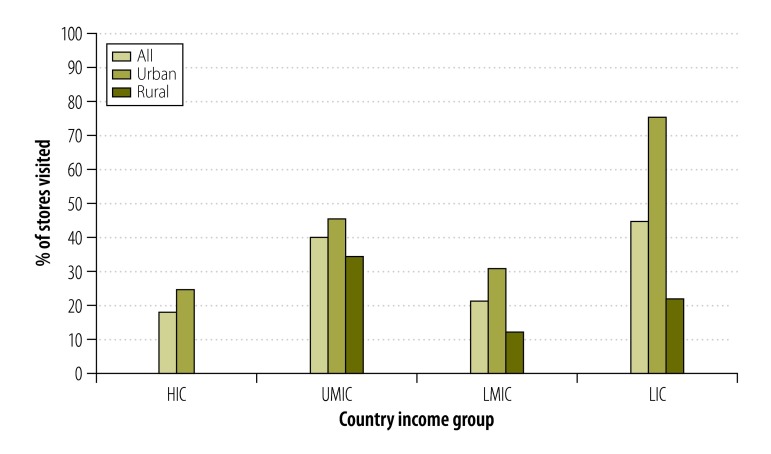
Proportion of tobacco-selling stores that had point-of-sale tobacco advertising, 16 countries, 2009–2012

HIC: high-income country; LIC: low-income country; LMIC: lower-middle-income country; UMIC: upper-middle-income country.

Note: Countries were categorized according to the World Bank’s 2006 classification.^11^

The percentage of tobacco-selling stores visited that had point-of-sale tobacco advertising did not appear to differ clearly by country income group: 18% (13/72) in high-income, 40% (48/120) in upper-middle-income, 21% (36/171) in lower-middle-income and 44% (42/95) in low-income countries (Fig. 4 and Table 2). However the percentages across all countries were generally higher in the urban communities (41%; 96/235) than in the rural communities (19%; 43/223).

### Self-reported data

Of the 11 842 interviewees, 5349 (45%; range: 4–100%) reported exposure to at least one type of tobacco marketing over the previous six months and 1184 (10%; range: 0–56%) reported exposure to at least five types of marketing over the same period (available from the corresponding author).

#### Pull marketing

##### Traditional

**Fig. 5:**
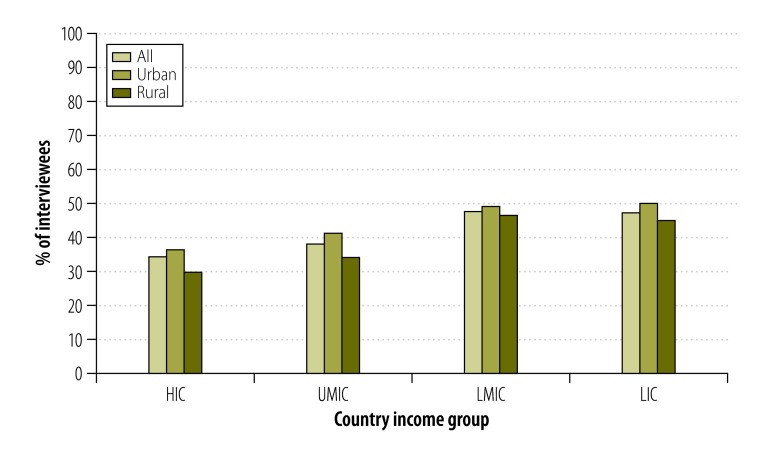
Proportion of urban or rural interviewees who reported seeing at least one traditional type of tobacco marketing in the previous six months, 16 countries, 2009–2012

HIC: high-income country; LIC: low-income country; LMIC: lower-middle-income country; UMIC: upper-middle-income country.

Notes: Countries were categorized according to the World Bank’s 2006 classification.^11^ Traditional types of marketing were posters, signage, television, radio, print and cinema.

Interviewees in high-income countries were least likely to report exposure to all forms of traditional marketing except print media, although differences between other country income groups varied by the type of marketing (Fig. 5; further details available from corresponding author). Overall, television marketing – seen by 3501 (30%) of interviewees in the previous six months – was the most common form of traditional marketing, followed by posters (2334; 20%), print media (1949; 16%), signage (1934; 16%), radio (1465; 12%) and cinema marketing (567; 5%). All forms of traditional marketing except television marketing – and exposure to at least one form of traditional marketing – were less common in rural communities than urban ones (Table 4 available at: http://www.who.int/bulletin/volumes/93/12/15-155846).

**Table 4:** Individuals who reported seeing tobacco marketing within the previous six months, 16 countries, 2009–2011

Country^a^	No. of individuals reporting seeing marketing/individuals interviewed (%)													
	Traditional marketing								Non-traditional marketing					
	Posters^b^	Signage^c^	Television	Radio	Print media^d^	Cinema	Seen at least one type		Sponsorship^e^	On other products^f^	Internet	Free samples	Vouchers^g^	Seen at least one type
**All countries**														
All communities	2335/11819 (19.8)	1934/11813 (16.4)	3501/11815 (29.6)	1465/11811 (12.4)	1949/11815 (16.5)	567/11813 (4.8)	5012/11820 (42.4)		1060/11818 (9.0)	1468/11818 (12.4)	938/11817 (7.9)	491/11816 (4.2)	491/11818 (4.2)	2139/11823 (18.1)
Urban	1332/5800 (23.0)	1164/5795 (20.1)	1625/5797 (28.0)	763/5793 (13.2)	1234/5796 (21.3)	351/5795 (6.1)	2538/5800 (43.8)		712/5799 (12.3)	950/5799 (16.4)	630/5799 (10.9)	293/5798 (5.1)	312/5799 (5.4)	1391/5804 (24.0)
Rural	1002/6019 (16.7)	770/6018 (12.8)	1876/6018 (31.2)	702/6018 (11.7)	715/6019 (11.9)	216/6018 (3.6)	2474/6020 (41.1)		348/6019 (5.8)	518/6019 (8.6)	308/6018 (5.1)	198/6018 (3.3)	179/6019 (3.0)	748/6019 (12.4)
**High-income countries**														
Canada														
Urban	59/807 (7.3)	67/807 (8.3)	68/807 (8.4)	17/807 (2.1)	162/807 (20.1)	18/807 (2.2)	244/807 (30.2)		108/807 (13.4)	54/807 (6.7)	39/807 (4.8)	4/807 (0.5)	7/807 (0.9)	165/807 (20.5)
Rural	27/338 (8.0)	23/338 (6.8)	29/338 (8.6)	9/338 (2.7)	62/338 (18.3)	12/338 (3.6)	95/338 (28.1)		35/338 (10.4)	15/338 (4.4)	14/338 (4.1)	0/338 (0.0)	3/338 (0.9)	55/338 (16.3)
Sweden														
Urban	66/495 (13.3)	97/491 (19.8)	48/492 (9.8)	4/490 (0.8)	194/492 (39.4)	19/491 (3.9)	237/495 (47.9)		44/493 (8.9)	125/493 (25.4)	81/491 (16.5)	4/492 (0.8)	7/493 (1.4)	182/495 (36.8)
Rural	2/84 (2.4)	6/84 (7.1)	6/84 (7.1)	0/84 (0.0)	36/84 (42.9)	2/84 (2.4)	40/84 (47.6)		5/84 (6.0)	15/84 (17.9)	9/84 (10.7)	3/84 (3.6)	0/84 (0.0)	22/84 (26.2)
United Arab Emirates														
Urban	2/26 (7.7)	2/26 (7.7)	1/26 (3.9)	1/26 (3.9)	1/26 (3.9)	2/26 (7.7)	2/26 (7.7)		1/26 (3.9)	1/26 (3.9)	2/26 (7.7)	0/26 (0.0)	0/26 (0.0)	2/26 (7.7)
Rural	5/63 (7.9)	1/63 (1.6)	4/63 (6.4)	1/63 (1.6)	4/63 (6.4)	0/63 (0.0)	9/63 (14.3)		0/63 (0.0)	1/63 (1.6)	1/63 (1.6)	0/63 (0.0)	0/63 (0.0)	1/63 (1.6)
Total														
All communities	161/1813 (8.9)	196/1809 (10.8)	156/1810 (8.6)	32/1808 (1.8)	459/1810 (25.4)	53/1809 (2.9)	627/1813 (34.6)		193/1811 (10.7)	211/1811 (11.7)	146/1809 (8.1)	11/1810 (0.6)	17/1811 (0.9)	427/1813 (23.6)
Urban	127/1328 (9.6)	166/1324 (12.5)	117/1325 (8.8)	22/1323 (1.7)	357/1325 (26.9)	39/1324 (3.0)	483/1328 (36.4)		153/1326 (11.5)	180/1326 (13.7)	122/1324 (9.2)	8/1325 (0.6)	14/1326 (1.1)	349/1328 (26.3)
Rural	34/485 (7.0)	30/485 (6.2)	39/485 (8.0)	10/485 (2.1)	102/485 (21.0)	14/485 (2.9)	144/485 (29.7)		40/485 (8.3)	31/485 (6.4)	24/485 (5.0)	3/485 (0.6)	3/485 (0.6)	78/485 (16.1)
**Upper-middle-income countries**														
Argentina														
Urban	16/171 (9.4)	16/171 (9.4)	36/171 (21.1)	0/171 (0.0)	15/171 (8.8)	0/171 (0.0)	49/171 (28.7)		4/171 (2.3)	1/171 (0.6)	1/171 (0.6)	0/171 (0.0)	0/171 (0.0)	6/171 (3.5)
Rural	6/373 (1.6)	7/373 (1.9)	86/373 (23.1)	2/373 (0.5)	24/373 (6.4)	0/373 (0.0)	103/373 (27.6)		6/373 (1.6)	0/373 (0.0)	2/373 (0.5)	0/373 (0.0)	0/373 (0.0)	8/373 (2.1)
Brazil														
Urban	15/202 (7.4)	8/202 (4.0)	37/202 (18.3)	8/202 (4.0)	23/202 (11.4)	3/202 (1.5)	56/202 (27.7)		6/202 (3.0)	2/202 (1.0)	7/202 (3.5)	1/202 (0.5)	1/202 (0.5)	13/202 (6.4)
Rural	32/185 (17.3)	1/185 (0.5)	34/185 (18.4)	2/185 (1.1)	5/185 (2.7)	0/185 (0.0)	64/185 (34.6)		0/185 (0.0)	0/185 (0.0)	0/185 (0.0)	0/185 (0.0)	0/185 (0.0)	0/185 (0.0)
Chile														
Urban	35/51 (68.6)	18/51 (35.3)	51/51 (100.0)	37/51 (72.6)	24/51 (47.1)	0/51 (0.0)	51/51 (100.0)		2/51 (3.9)	39/51 (76.5)	9/51 (17.7)	0/51 (0.0)	0/51 (0.0)	39/51 (76.5)
Rural	3/76 (4.0)	1/76 (1.3)	12/76 (15.8)	4/76 (5.3)	3/76 (4.0)	0/76 (0.0)	14/76 (18.4)		0/76 (0.0)	2/76 (2.6)	0/76 (0.0)	0/76 (0.0)	0/76 (0.0)	2/76 (2.6)
Malaysia														
Urban	260/591 (44.0)	194/591 (32.8)	271/591 (45.9)	223/591 (37.7)	251/591 (42.5)	67/591 (11.3)	300/591 (50.8)		191/591 (32.3)	191/591 (32.3)	230/591 (38.9)	80/591 (13.5)	101/591 (17.1)	262/591 (44.3)
Rural	202/577 (35.0)	163/577 (28.3)	216/577 (37.4)	173/577 (30.0)	181/577 (31.4)	41/577 (7.1)	227/577 (39.3)		123/577 (21.3)	146/577 (25.3)	161/577 (27.9)	72/577 (12.5)	66/577 (11.4)	178/577 (30.9)
Poland														
Urban	6/26 (23.1)	5/26 (19.2)	4/26 (15.4)	1/26 (3.9)	6/26 (23.1)	1/26 (3.9)	12/26 (46.2)		2/26 (7.7)	3/26 (11.5)	4/26 (15.4)	4/26 (15.4)	2/26 (7.7)	11/26 (42.3)
Rural	10/63 (15.9)	9/63 (14.3)	7/63 (11.1)	1/63 (1.6)	8/63 (12.7)	1/63 (1.6)	23/63 (36.5)		1/63 (1.6)	5/63 (7.9)	3/63 (4.8)	1/63 (1.6)	0/63 (0.0)	8/63 (12.7)
South Africa														
Urban	48/98 (49.0)	44/98 (44.9)	54/98 (55.1)	45/98 (45.9)	53/98 (54.1)	21/98 (21.4)	80/98 (81.6)		30/99 (30.3)	29/99 (29.3)	17/99 (17.2)	30/99 (30.3)	34/99 (34.3)	50/99 (50.5)
Rural	38/95 (40.0)	33/95 (34.7)	34/95 (35.8)	46/95 (48.4)	29/95 (30.5)	3/94 (3.2)	65/95 (68.4)		16/95 (16.8)	17/95 (17.9)	5/94 (5.3)	16/94 (17.0)	10/95 (10.5)	25/95 (26.3)
Turkey														
Urban	124/795 (15.6)	127/795 (16.0)	170/795 (21.4)	45/795 (5.7)	89/795 (11.2)	12/795 (1.5)	252/795 (31.7)		43/795 (5.4)	87/795 (10.9)	21/795 (2.6)	6/795 (0.8)	4/795 (0.5)	110/795 (13.8)
Rural	30/412 (7.3)	40/412 (9.7)	85/412 (20.6)	19/412 (4.6)	31/412 (7.5)	6/412 (1.5)	113/412 (27.4)		14/412 (3.4)	39/412 (9.5)	11/412 (2.7)	3/412 (0.7)	0/412 (0.0)	56/412 (13.6)
Total														
All communities	825/3715 (22.2)	666/3715 (17.9)	1097/3715 (29.5)	606/3715 (16.3)	742/3715 (20.0)	155/3714 (4.2)	1409/3715 (37.9)		438/3716 (11.8)	561/3716 (15.1)	471/3715 (12.7)	213/3715 (5.7)	218/3716 (5.9)	768/3716 (20.7)
Urban	504/1934 (26.1)	412/1934 (21.3)	623/1934 (32.2)	359/1934 (18.6)	461/1934 (23.8)	104/1934 (5.4)	800/1934 (41.4)		278/1935 (14.4)	352/1935 (18.2)	289/1935 (14.9)	121/1935 (6.3)	142/1935 (7.3)	491/1935 (25.4)
Rural	321/1781 (18.0)	254/1781 (14.3)	474/1781 (26.6)	247/1781 (13.9)	281/1781 (15.8)	51/1780 (2.9)	609/1781 (34.2)		160/1781 (9.0)	209/1781 (11.7)	182/1780 (10.2)	92/1780 (5.2)	76/1781 (4.3)	277/1781 (15.6)
**Lower-middle-income countries**														
China														
Urban	329/1217 (27.0)	223/1216 (18.3)	527/1217 (43.3)	225/1215 (18.5)	158/1893 (18.4)	102/1216 (8.4)	636/1217 (52.3)		141/1217 (11.6)	263/1217 (21.6)	188/1219 (15.4)	81/1217 (6.7)	73/1217 (6.0)	372/1220 (30.5)
Rural	234/1893 (12.4)	135/1892 (7.1)	833/1892 (44.0)	265/1892 (14.0)	224/1216 (8.4)	52/1893 (2.8)	946/1894 (50.0)		44/1893 (2.3)	171/1893 (9.0)	83/1893 (4.4)	35/1893 (1.9)	34/1893 (1.8)	261/1893 (13.8)
Colombia														
Urban	89/151 (58.9)	67/151 (44.4)	88/151 (58.3)	68/151 (45.0)	53/151 (35.1)	9/151 (6.0)	115/151 (76.2)		58/151 (38.4)	60/151 (39.7)	11/151 (7.3)	53/151 (35.1)	54/151 (35.8)	64/151 (42.4)
Rural	96/127 (75.6)	79/127 (62.2)	89/127 (70.1)	72/127 (56.7)	55/127 (43.3)	9/127 (7.1)	108/127 (85.0)		66/127 (52.0)	67/127 (52.8)	17/127 (13.4)	62/127 (48.8)	64/127 (50.4)	70/127 (55.1)
Iran (Islamic Republic of)														
Urban	17/321 (5.3)	50/321 (15.6)	11/321 (3.4)	0/321 (0.0)	9/321 (2.8)	22/321 (6.9)	76/321 (23.7)		3/321 (0.9)	7/321 (2.1)	1/321 (0.3)	0/321 (0.0)	1/321 (0.3)	12/321 (3.7)
Rural	1/272 (0.4)	5/272 (1.8)	2/272 (0.7)	2/272 (0.7)	2/272 (0.7)	4/272 (1.5)	11/272 (4.0)		1/272 (0.4)	1/272 (0.4)	1/272 (0.4)	0/272 (0.0)	0/272 (0.0)	1/272 (0.4)
Total														
All communities	766/3981 (19.2)	559/3979 (14.1)	1550/3980 (38.9)	632/3978 (15.9)	501/3980 (12.6)	198/3980 (5.0)	1892/3982 (47.5)		313/3981 (7.9)	569/3981 (14.3)	301/3983 (7.6)	231/3981 (5.8)	226/3981 (5.7)	780/3984 (19.6)
Urban	435/1689 (25.8)	340/1688 (20.1)	626/1689 (37.1)	293/1687 (17.4)	286/1688 (16.9)	133/1688 (7.9)	827/1689 (49.0)		202/1689 (12.0)	330/1689 (19.5)	200/1691 (11.8)	134/1689 (7.9)	128/1689 (7.6)	448/1692 (26.5)
Rural	331/2292 (14.4)	219/2291 (9.6)	924/2291 (40.3)	339/2291 (14.8)	215/2292 (9.4)	65/2292 (2.8)	1065/2293 (46.5)		111/2292 (4.8)	239/2291 (10.4)	101/2291 (4.4)	97/2292 (4.2)	98/2292 (4.3)	332/2292 (14.5)
**Low-income countries**														
India														
Urban	211/766 (27.6)	187/766 (24.4)	201/766 (26.2)	57/766 (7.4)	94/766 (12.3)	63/766 (8.2)	353/766 (46.1)		47/766 (6.1)	57/766 (7.4)	3/766 (0.4)	19/766 (2.5)	22/766 (2.9)	62/766 (8.1)
Rural	254/1352 (18.8)	189/1352 (14.0)	396/1352 (29.3)	53/1352 (3.9)	76/1352 (5.6)	83/1352 (6.1)	561/1352 (41.5)		14/1352 (1.0)	17/1352 (1.3)	0/1352 (0.0)	2/1352 (0.2)	1/1352 (0.1)	29/1352 (2.1)
Pakistan														
Urban	33/57 (57.9)	44/57 (77.2)	36/57 (63.2)	26/57 (45.6)	23/57 (40.4)	9/57 (15.8)	50/57 (87.7)		19/57 (33.3)	14/57 (24.6)	16/57 (28.1)	9/57 (15.8)	5/57 (8.8)	23/57 (40.4)
Rural	23/54 (42.6)	35/54 (64.8)	26/54 (48.2)	10/54 (18.5)	10/54 (18.5)	1/54 (1.9)	42/54 (77.8)		3/54 (5.6)	1/54 (1.9)	0/54 (0.0)	2/54 (3.7)	1/54 (1.9)	5/54 (9.3)
Zimbabwe														
Urban	22/26 (84.6)	15/26 (57.7)	22/26 (84.6)	6/26 (23.1)	13/26 (50.0)	3/26 (11.5)	25/26 (96.2)		13/26 (50.0)	17/26 (65.4)	0/26 (0.0)	2/26 (7.7)	1/26 (3.9)	18/26 (69.2)
Rural	39/55 (70.9)	43/55 (78.2)	17/55 (30.9)	43/55 (78.2)	31/55 (56.4)	2/55 (3.6)	53/55 (96.4)		20/55 (36.4)	21/55 (38.2)	1/55 (1.8)	2/55 (3.6)	0/55 (0.0)	27/55 (49.1)
Total														
All communities	582/2310 (25.2)	513/2310 (22.2)	698/2310 (30.2)	195/2310 (8.4)	247/2310 (10.7)	161/2310 (7.0)	1084/2310 (46.9)		116/2310 (5.0)	127/2310 (5.5)	20/2310 (0.9)	36/2310 (1.6)	30/2310 (1.3)	164/2310 (7.1)
Urban	266/849 (31.3)	246/849 (29.0)	259/849 (30.5)	89/849 (10.5)	130/849 (15.3)	75/849 (8.8)	428/849 (50.4)		79/849 (9.3)	88/849 (10.4)	19/849 (2.2)	30/849 (3.5)	28/849 (3.3)	103/849 (12.1)
Rural	316/1461 (21.6)	267/1461 (18.3)	439/1461 (30.1)	106/1461 (7.3)	117/1461 (8.0)	86/1461 (5.9)	656/1461 (44.9)		37/1461 (2.5)	39/1461 (2.7)	1/1461 (0.1)	6/1461 (0.4)	2/1461 (0.1)	61/1461 (4.2)

^a^ Countries were categorized according to the World Bank’s 2006 classification.^11^

^b^ For example billboards, pasted on walls, visible on the sides of taxis and buses.

^c^ Permanently sponsored signage on shops or other buildings.

^d^ For example newspapers and magazines.

^e^ Sponsorship of sporting, music or other events.

^f^ On products such as umbrellas, ashtrays, shopping bags, clothing or any other products.

^g^ Promotional vouchers that allow discounts.

The likelihood that interviewees from low-income countries reported exposure to at least one form of traditional marketing was almost 10 times higher (OR: 9.77; 95% CI: 1.24–76.77) than in high-income countries. Specifically, the likelihood of exposure to radio (OR: 46.05; 95% CI: 1.29–1642.57), signage (OR: 11.02; 95% CI: 1.07–113.60), television (OR: 9.42; 95% CI: 1.21–73.20) and cinema marketing of tobacco (OR: 3.08; 95% CI: 1.46–6.49) were significantly higher in low-income than in high-income countries (Table 5). Compared with the interviewees from urban communities, the likelihood that interviewees from rural communities reported exposure to traditional marketing was either significantly lower – posters, signage, print and cinema marketing – or not significantly different – television and radio marketing (Table 5).

**Table 5:** The likelihood that interviewees reported seeing traditional types of tobacco marketing within the previous six months, 16 countries, 2009–2012

Group	OR (95% CI)^a^						
	Posters	Signage	Television	Radio	Print media	Cinema	Any type
**Community type**							
Urban	1	1	1	1	1	1	1
Rural	0.41 (0.28–0.59)	0.34 (0.24–0.48)	0.86 (0.62–1.21)	0.64 (0.40–1.02)	0.54 (0.39–0.75)	0.49 (0.30–0.78)	0.72 (0.53–0.98)
**Country income group^b^**							
High	1	1	1	1	1	1	1
Upper-middle	2.19 (0.28–16.87)	1.29 (0.18–9.03)	4.19 (0.77–22.84)	9.50 (0.46–195.60)	0.75 (0.12–4.53)	0.70 (0.33–1.50)	1.57 (0.29–8.49)
Lower-middle	2.37 (0.22–24.86)	2.16 (0.23–20.09)	3.73 (0.54–26.00)	13.89 (0.42–454.42)	0.43 (0.05–3.45)	1.63 (0.81–3.27)	2.19 (0.32–15.17)
Low	11.05 (0.94–129.43)	11.02 (1.07–113.60)	9.42 (1.21–73.20)	46.05 (1.29–1642.57)	1.29 (0.15–11.22)	3.08 (1.46–6.49)	9.77 (1.24–76.77)

CI: confidence interval; OR: odds ratio.

^a^ Derived from logistic multilevel regression models.

^b^ Countries were categorized according to the World Bank’s 2006 classification.^11^

##### Non-traditional

**Fig. 6:**
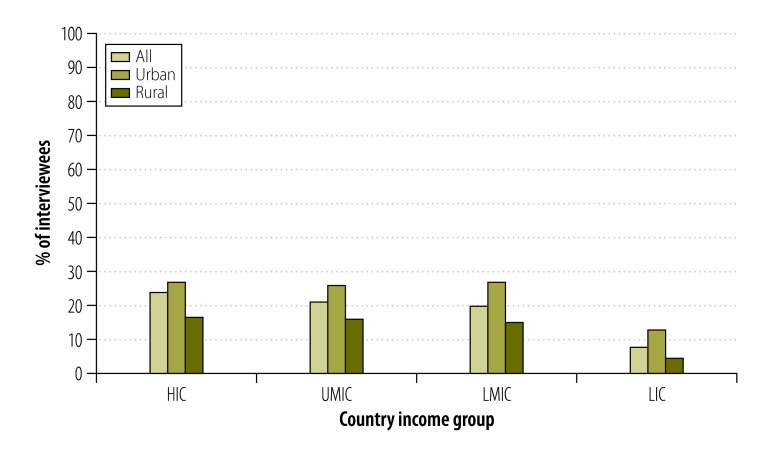
Proportion of urban or rural interviewees who reported seeing at least one non-traditional type of tobacco marketing in the previous six months, 16 countries, 2009–2012

HIC: high-income country; LIC: low-income country; LMIC: lower-middle-income country; UMIC: upper-middle-income country.

Notes: Countries were categorized according to the World Bank’s 2006 classification.^11^ Non-traditional types of marketing were sponsorship, tobacco marketing on other products, on the internet, free samples and vouchers.

Non-traditional marketing was reported less frequently than traditional marketing (Table 4). Although tobacco marketing on other products – e.g. umbrellas – was the most commonly reported form of non-traditional marketing, only 1468 (12%) of the interviewees reported seeing such marketing in the previous six months (Fig. 6 and Table 4). Country income group appeared to have little impact on exposure to non-traditional marketing but overall exposure and exposure to each form of non-traditional marketing appeared more common in the urban communities than in the rural.

After controlling for confounders, the likelihood of exposure to non-traditional tobacco marketing in the low- and middle-income countries appeared similar to that in the high-income countries (Table 6). However, compared with their urban counterparts, the likelihood that rural interviewees reported exposure to one or more forms of non-traditional marketing was significantly lower (OR: 0.38; 95% CI: 0.25–0.59) – including the odds of exposure to sponsorship (OR: 0.35; 95% CI: 0.22–0.56), marketing on other products (OR: 0.32; 95% CI: 0.20–0.54), internet marketing (even after controlling for internet access; OR: 0.45; 95% CI: 0.26–0.78), free samples (OR: 0.37; 95% CI: 0.21–0.66) and vouchers (OR: 0.28; 95% CI: 0.16–0.51).

**Table 6:** The likelihood that interviewees reported seeing non-traditional types of tobacco marketing within the previous six months, 16 countries, 2009–2012

Group	OR (95% CI)^a^					
	Sponsorship	On other products	Internet	Free samples	Vouchers	Any type
**Community type**						
Urban	1	1	1	1	1	1
Rural	0.35 (0.22–0.56)	0.32 (0.20–0.54)	0.45 (0.26–0.78)	0.37 (0.21–0.66)	0.28 (0.16–0.51)	0.38 (0.25–0.59)
**Country income group^b^**						
High	1	1	1	1	1	1
Upper-middle	0.57 (0.04–7.71)	0.59 (0.03–12.56)	0.75 (0.06–8.66)	4.03 (0.07–224.84)	1.94 (0.04–88.53)	0.82 (0.07–10.03)
Lower-middle	0.91 (0.05–18.13)	1.26 (0.04–42.87)	0.46 (0.03–7.76)	10.20 (0.11–987.76)	10.73 (0.15–774.21)	0.96 (0.05–17.18)
Low	1.32 (0.06–29.21)	1.10 (0.03–42.45)	0.06 (0.00–1.47)	10.95 (0.11–1086.21)	1.19 (0.01–120.60)	1.03 (0.05–20.59)

CI: confidence interval; OR: odds ratio.

^a^ Derived from logistic multilevel regression models.

^b^ Countries were categorized according to the World Bank’s 2006 classification.^11^

## Discussion

Our study has three important findings in relation to tobacco marketing. First, we identified high levels of ongoing exposure to tobacco marketing – despite 14 of the study countries having ratified the FCTC at the time the data were collected; by December 2014, Argentina had signed but not ratified the FCTC and Zimbabwe had only acceded to it. Although ratification requires countries to implement comprehensive marketing bans, 10% of the interviewees reported seeing at least five types of tobacco marketing in the six months before interview and 45% reported seeing at least one type of tobacco marketing over the same period. Second, we detected substantially higher levels of tobacco marketing in the lower-income countries we investigated than in the higher-income. This result is consistent with the tobacco industry specifically targeting low- and middle-income countries,^20^^,^^21^ which could be due to large youth populations in lower-income countries and to high-income countries having more established policies on tobacco control.^22^ Third, for 13 of 15 marketing measures, exposure was significantly lower in the rural communities than in the urban ones.

High levels of tobacco marketing may reflect failure to enact legislation and/or to enforce compliance.^23^ Yet many of our interviewees – even those from countries with highly regarded tobacco control measures such as Brazil, Canada and Sweden^24^^–^^26^ – reported substantial exposure to tobacco marketing. This indicates that the tobacco industry may still be finding ways to market its products. Given that we recorded 10 times greater exposure to traditional marketing in the low-income countries than in the high-income countries – but similar levels of exposure to non-traditional marketing across all country income groups – it appears that legislation may have been relatively successful in controlling traditional marketing in high-income countries. This success may have resulted in the tobacco industry using newer, less regulated forms of marketing. Therefore, enforcement may need to be stronger and legislation continuously adapted to the changing marketing practices of the tobacco industry. Data on the tobacco industry’s marketing expenditure would also be useful, but such data are available for very few countries^27^ and not for any of our study countries.

Our observation of more intense tobacco marketing in urban communities than in rural communities is consistent with evidence that the tobacco industry focuses its marketing and distribution on areas with the greatest potential impact – i.e. areas with dense populations^28^^,^^29^ that can be easily reached at relatively low cost.^30^

Our study had several limitations. First, although diverse,^11^ the countries studied are not necessarily representative of low-, middle- and high-income countries globally and the communities investigated within each country are not necessarily representative of all communities.^10^ Although this means that the results cannot reliably be extrapolated to all communities within a country, the demographic characteristics of our interviewees do appear to match those of adults in the corresponding national populations.^11^ We also note that the main tobacco company in two of the three lower-middle-income countries – i.e. China and the Islamic Republic of Iran – is state-owned.^31^ Countries with state-owned monopolies traditionally do not market their products aggressively because the lack of competition renders this unnecessary.^32^ Our findings, especially those on self-reported marketing, indicate that the tobacco marketing environment may well be affected by state ownership of the local tobacco industry. In the Islamic Republic of Iran, for example, exposure to most forms of marketing appeared to be less intense than in other lower-middle-income countries. Our results appear to be consistent with data from WHO’s Global Adult Tobacco Survey^33^ that was conducted in 16 countries, including six of our study countries – Brazil, China, India, Pakistan, Poland and Turkey. Although the WHO’s survey did not include statistical comparisons, it did show relatively high self-reported exposure to tobacco marketing in lower-income countries – with the exception of the Russian Federation – and in urban communities.^33^ Our findings also seem similar to those from the International Tobacco Control Policy Evaluation Project,^34^ which has collected data from 22 countries, including five of our study countries – Brazil, Canada, China, India and Malaysia.

Second, the sample size varied markedly by country – both for the number of communities and number of interviewees. We would expect more uncertainty in an estimate for a country in which only a few communities are sampled. Additionally, the number of countries per country income group and the small number of communities surveyed in two of the three low-income countries may explain the wide CIs seen in some significant comparisons between low- and high-income countries. Third, although the methods used have been shown to be reliable,^9^ only one tobacco-selling store was visited per community during the walk – and it is not possible to know whether the selected store was representative of all stores within the community. Fourth, our study was limited by difficulties in estimating the tobacco industry’s marketing expenditure in each study country and by exposure of many individuals to cross-border marketing – including internet marketing. Finally, the study used data collected between 2009 and 2012 and some of the countries have since taken further steps to strengthen their tobacco marketing regulations.

Our study also has strengths. The Environmental Profile of a Community’s Health study takes a comprehensive approach to data collection, using both direct observation and self-reported data to assess the level and nature of diverse forms of tobacco marketing at both community and individual level; an approach shown to be reliable.^9^ The countries included in our analysis are very diverse in terms of both economics and culture. Additionally, although differences in self-reported exposure to marketing will reflect access to certain types of media, we were able to control for internet access and television and radio ownership in the individual-level models.

This study indicates that tobacco marketing remains ubiquitous even in countries that have ratified the FCTC. Given the strength of the link between marketing by the tobacco industry and the prevalence of smoking,^2^^–^^4^ there is an urgent need for countries either to implement comprehensive controls on tobacco marketing or to enforce such controls more effectively.
